# The Transcription Factor T-Bet Is Regulated by MicroRNA-155 in Murine Anti-Viral CD8^+^ T Cells *via* SHIP-1

**DOI:** 10.3389/fimmu.2017.01696

**Published:** 2017-12-06

**Authors:** Jennifer L. Hope, Christopher J. Stairiker, Panagiota I. Spantidea, Donald T. Gracias, Alison J. Carey, Adam J. Fike, Marjan van Meurs, Inge Brouwers-Haspels, Laurine C. Rijsbergen, Joseph A. Fraietta, Yvonne M. Mueller, Rosemarieke C. Klop, Erietta Stelekati, E. John Wherry, Stefan J. Erkeland, Peter D. Katsikis

**Affiliations:** ^1^Department of Immunology, Erasmus MC University Medical Center, Rotterdam, Netherlands; ^2^Department of Microbiology and Immunology, Drexel University College of Medicine, Philadelphia, PA, United States; ^3^Department of Pediatrics, Drexel University College of Medicine, Philadelphia, PA, United States; ^4^Center for Cellular Immunotherapies and Abramson Cancer Center, University of Pennsylvania, Philadelphia, PA, United States; ^5^Institute for Immunology, University of Pennsylvania, Philadelphia, PA, United States

**Keywords:** MicroRNAs, CD8^+^ T cells, T-bet, influenza, Src homology 2-containing inositol phosphatase-1, miR-155

## Abstract

We report here that the expression of the transcription factor T-bet, which is known to be required for effector cytotoxic CD8^+^ T lymphocytes (CTL) generation and effector memory cell formation, is regulated in CTL by microRNA-155 (miR-155). Importantly, we show that the proliferative effect of miR-155 on CD8^+^ T cells is mediated by T-bet. T-bet levels in CTL were controlled *in vivo* by miR-155 *via* SH2 (Src homology 2)-containing inositol phosphatase-1 (SHIP-1), a known direct target of miR-155, and SHIP-1 directly downregulated T-bet. Our studies reveal an important and unexpected signaling axis between miR-155, T-bet, and SHIP-1 in *in vivo* CTL responses and suggest an important signaling module that regulates effector CTL immunity.

## Introduction

Cytotoxic CD8^+^ T cells (CTLs) are an essential component of the adaptive immune system, responsible for the clearance or control of cells infected with viruses or intracellular bacteria and tumors (1, 2). Identifying the mechanisms that control the formation of effector and memory CTL is essential for rational vaccine design and immunotherapeutic approaches. Transcription factors have been recognized as crucial elements that control and direct the fate of CTL toward effector or memory differentiation states (3, 4). In particular, the transcription factors T-bet, Eomes, Blimp-1, and Bcl-6 have been shown to regulate the differentiation and function of CTL (5). T-bet- and Eomes-deficient mice have shown that the “graded expression” of these transcription factors regulate the early effector and memory phenotype of CTL in response to infection with lymphocytic choriomeningitis virus (LCMV) (3). Specifically, T-bet has been linked to the generation of effector and effector memory CD8^+^ T cells (6–8) and the production of the pro-inflammatory cytokine IFNγ by CTL (6, 7). Therefore, understanding how T-bet expression is regulated in CTL is critical for our understanding of the pathways that control effector and memory CTL formation.

MicroRNAs (miRNAs) are ~22 nucleotides small, single-stranded non-coding RNAs that are important post-transcriptional regulators of gene expression and play essential roles in modulating key cellular processes (9–11). miRNAs have been shown to control CD8^+^ T cell formation, differentiation, and function. In the absence of miRNA’s, CD8^+^ T cells fail to develop normally (12) or aberrantly respond *in vivo* to *Listeria monocytogenes* infection (13). However, the regulatory role of specific miRNAs in generating effective CTL responses to viral infection and tumors is only now being elucidated. Indeed, the regulatory effect of miRNAs is not only cell-type specific, but also context dependent and influenced by the activation state of the cell and *in vivo* inflammatory environment (14). Thus, miRNA expression is expected to change depending on the differentiation and activation state of the CTL and this could also be influenced by the milieu and location in which the CTL response is elicited. Previous *in vitro* studies have indicated that miRNA expression in CTL changes with differentiation (15) and we have shown that *in vivo* miR-155 expression levels dynamically change during differentiation from naïve to effector to memory CTL (16).

We, and others, have demonstrated that, in the absence of miR-155, effector CTL responses against acute infections with influenza A virus or *L. monocytogenes*, and tumors are severely diminished (16–19). Furthermore, in the absence of miR-155, the generation of memory CTL is decreased (16). This suggested that overexpression of miR-155 could enhance CTL responses to infections and cancer; indeed, we (16) and others (20) have found that overexpressing miR-155 in CD8^+^ T cells causes significant expansion. Multiple mechanisms and targets by which miR-155-deficiency can affect CTL responses have been proposed including type-I IFN and STAT1/2 signaling (16), γc chain cytokine signaling (20), and SOCS-1 (14, 17), which suggests that miR-155 regulation of CTL is context dependent (14). This also raises the question of whether the overexpression of a specific miRNA targets the same mRNA as steady-state endogenous miRNA. Because miRNA and target mRNA availability is a dynamic process (21, 22), it is likely that supranormal levels of a miRNA may allow the targeting of additional mRNA which are normally outcompeted by other targets.

Increasing miR-155 expression in CTL augmented anti-viral effector CTL and skewed memory CD8^+^ T cells toward an effector memory phenotype. miR-155 overexpression induced enhanced T-bet expression and downregulated the inhibitory phosphatase SH2 (Src homology 2)-containing inositol phosphatase-1 (SHIP-1). T-bet upregulation was necessary for effector CTL augmentation by miR-155. Importantly, we show that SHIP-1 regulated T-bet expression and promoted the effector responses in miR-155-overexpressing CTL. Thus, T-bet expression can be controlled by miR-155 *via* SHIP-1 signaling and we have revealed a novel regulatory pathway for T-bet expression as well as effector and memory CTL generation.

## Materials and Methods

### Animals and Infections

C57BL/6 Tg(TcraTcrb)1100Mjb/J (OT-I) were backcrossed with B6.SJL-Ptprca Pepcb/BoyJ (CD45.1^+^) mice (both from the Jackson Laboratory) to generate OT-I CD45.1^+^ mice on the C57BL6/J background. C57BL/6J mice, miR-155-deficient OT-I mice, and T-bet^+/‒^ OT-I mice (on the C57BL/6J background) were kept in a barrier facility (certified by the Association for the Assessment and Accreditation of Laboratory Animal Care) at Drexel University College of Medicine, or in a barrier facility at Erasmus University Medical Center. This study was carried out in accordance with the recommendations of the Institutional Animal Care and Use Committee (IACUC) or the Instantie voor Dierenwelzijn (IvD). The protocols were approved by the IACUC or IvD. Female mice 8–10 weeks old were anesthetized with 2.5% isoflurane gas and were infected intranasally with influenza virus strain A/WSN/33-expressing OVA_(257–264)_ (WSN-OVA, a gift from DJ Topham, University of Rochester Medical Center).

### Adoptive Transfer Experiments

Equal numbers of sorted Thy1.1^+^ or GFP^+^ OT-I CD45.1^+^ cells (1 × 10^4^) were intravenously transferred into CD45.2^+^ C57BL/6J wild-type recipient mice. Three h later, the recipient mice were anesthetized using 2.5% isoflurane gas and infected intranasally with influenza virus WSN-OVA.

### Retroviral Production

The miR-155-encoding region from the MigR1-miR-155-GFP vector (16) was cloned into the MSCV-IRES-Thy1.1 vector (provided by P. Marrack, University of Colorado). A scrambled control insert producing no functional miRNA was similarly cloned into the MSCV-IRES-Thy1.1 vector. The PINCO-empty vector-GFP, PINCO-SHIP-1-overexpressing-GFP, and PINCO-D675A-SHIP-1 (SHIP-1 dominant negative) retroviral vectors were a kind gift from Dr. M. Caligiuri (Ohio State University) (23). Retroviruses were produced in the Platinum-E cell line (Cell Biolabs, San Diego, CA, USA).

### CD8^+^ T-Cell Isolation and Retroviral Transduction

Retroviral transduction of primary OT-I CD8^+^ T cells was completed as previously described (16). Splenic CD8^+^ T cells were isolated by negative selection with magnetic beads (EasySep; Stemcell Technologies) from uninfected OT-I CD45.1^+^ female mice 8–10 weeks old. The purity of CD8^+^ T cells was >90% as determined by flow cytometry. Isolated CD8^+^ T cells were activated for 48 h using solid-phase α-CD3 (0.25 µg/mL, clone: 17A2; eBioscience, San Diego, CA, USA) and α-CD28 antibodies (5 µg/mL, clone: 37.51; eBioscience) in 10% RPMI medium with 20 U/mL recombinant human IL-2 (Roche, Switzerland), 5 ng/mL of recombinant murine IL-7 and 5 ng/mL recombinant murine IL-15 (both from PeproTech, Rocky Hill, NJ, USA). Cells were collected and plated at a density of 3 × 10^6^ cells per 2 mL in poly-d-lysine plates (ThermoFisher, Waltham, MA, USA) coated with 20 µg/mL of Retronectin (Takara, Japan) and pre-loaded with retroviral supernatants. Cells were incubated for an additional 48 h. Transduction efficiency was determined by expression of GFP or Thy1.1. Transduced cells were sorted with an FACS Aria III sorter (BD Biosciences, San Jose, CA, USA). Overexpression of miR-155 was confirmed by TaqMan miRNA gene expression quantitative real-time polymerase chain reaction (qRT-PCR) (ThermoFisher) and was determined to be ~5-fold increased over control-transduced cells (Figure S1G in Supplementary Material).

### Flow Cytometry

Cells were stained as previously described (24). In all stains, cells were pretreated with anti-CD16/32 (Fc Block; 2.4G2; BioLegend, San Diego, CA, USA) for 15 min before continuing with surface staining. For surface stains, cells were stained for 20 min on ice. Cells were stained with the following fluorochrome conjugated monoclonal antibodies: CD8a (clone 53-6.7), CD45.1 (clone A20), CD45.2 (clone 104), Thy1.1 (clone HIS51) (all from eBioscience), CD25 (clone PC61), CD69 (clone H1.2F3), CD44 (clone 1M7), CD62L (clone MEL-14) (all from BD Bioscience, San Jose, CA, USA), KLRG1 (clone 2F1/KLRG1), IL-7R/CD127 (clone A7R34), and PD-1 (clone 29F-IAI2) (all from BioLegend). Cells were also stained with Cy5.5-labeled Annexin V (BD Biosciences) and APC-labeled tetramers of H-2^b^ major histocompatibility complex class I loaded with OVA_(257–264)_. After staining, cells were washed two times with HBSS containing 3% FBS and 0.02% sodium azide and fixed with 1% PFA. For Annexin V staining, all buffers contained 2.5 mM CaCl_2_. For staining of intracellular cytokines, cells were stimulated with SIINFEKL peptide for 6 h at 37°C, 5% CO_2_ in the presence of GolgiPlug (BD Biosciences) and monoclonal antibody against CD107a (clone ID4B) or isotype control. Cells were surface stained as above including fluorochrome conjugated monoclonal antibody against CD107a (clone ID4B) or the appropriate isotype control (both BioLegend), then fixed overnight at 4°C with IC Fixation Buffer, washed using Perm/Wash buffer (eBioscience) and stained for intracellular cytokines for 45 min at 4°C. Fluorochrome-conjugated anti-IFNγ monoclonal antibody (clone XMG1.2), anti-TNF-α monoclonal antibody (clone MP6-XT22), or the appropriate isotype controls (all from eBioscience) were used for intracellular stains. After staining, cells were washed twice with Perm/Wash buffer (eBioscience) and fixed with 1% PFA. For staining of transcription factors, cells were surface stained as above then fixed for 1 h at 4°C with FoxP3 Fixation Buffer, washed using Perm/Wash buffer (eBioscience), and stained for transcription factors for 1 h at 4°C. The following antibodies were used in combination with intracellular flow cytometry: anti-T-bet antibody (clone 4B10, BioLegend), anti-Eomes antibody (clone DAN11MAG, eBioscience), or the appropriate isotype controls. After staining, cells were washed twice with Perm/Wash buffer (eBioscience) and fixed with 1% PFA. Anti-T-bet antibody staining was titrated using wild-type (T-bet^+/+^) and T-bet^+/‒^ splenocytes to achieve clear distinction between heterozygote and homozygote T-bet expression (Figure S3 in Supplementary Material). For staining of SHIP-1, cells were surface stained as above, then fixed using Fixation Buffer (BioLegend) for 20 min at RT, washed with Perm/Wash Buffer (BioLegend), and stained for 20 min at RT. The following antibodies were used in combination with intracellular flow cytometry: anti-SHIP-1 antibody (clone P1C1-A5). After staining, cells were washed twice with Perm/Wash buffer (BioLegend) and fixed with 1% PFA. All samples were collected with an LSR-Fortessa (BD Biosciences) and analyzed with FlowJo software (Treestar, Ashland, OR, USA).

### Quantitative Real-time PCR

For *ex vivo* measurement of miR-155, SHIP-1 (*Inpp5d*), T-bet (*Tbx21*), Eomes (*Eomes*), Blimp-1 (*Prdm1*), Bcl-6, (*Bcl6*) Granzyme B (*gzmb*) and PD-1 (*Pdcd1*), donor OT-I CD8^+^ T cells were sorted from the lungs or spleens of influenza virus-infected animals at the indicated time points. Total RNA, including miRNA, was extracted using the miRNeasy mini kit (Qiagen, Germantown, MD, USA) as per manufacturer’s instructions. cDNA was synthesized from 100-ng RNA with the high-capacity cDNA Reverse Transcription Assay (ThermoFisher). The expression of miR-155 was measured by qRT-PCR with the TaqMan mmu-miR-155-5p miRNA Assay (ThermoFisher). The expression of snoRNA-429 served as endogenous control. Primers for *Inpp5d, Tbx21, Eomes, Prdm1, Bcl6, gzmb*, and *Pdcd1* (TaqMan gene expression assays all from ThermoFisher) were used to detect expression of these genes by qRT-PCR. Expression was normalized to GAPDH expression. All assays were run using a 7900 HT RT-PCR System. Expression was evaluated by the comparative cycling threshold (ΔΔ*C_T_*) method.

### Western Blot

Donor OT-I CD8^+^ T cells were sorted from the lungs of wild-type recipient mice 9 days post-influenza virus infection, washed two times with 1X phosphate-buffered saline (PBS) and cell pellets were stored at −20°C in RIPA buffer (150-mM sodium chloride, 1.0% NP-40, 0.5% sodium deoxycholate, 0.1% sodium dodecyl sulfate, 50 mM Tris, pH 8.0). Samples were freeze-thawed three times and then heated to 99°C on a heat block for 10 min. Samples were loaded into a 4–12% Mini-PROTEAN TGX Gel (Bio-Rad, Hercules, CA, USA), then transferred at 4°C to PVDF membrane (EMD Millipore, Darmstadt, Germany). Membranes were blocked at room temperature for 1 h in 5% BSA in Tris-buffered saline with tween (TBST), then incubated overnight at 4°C with primary antibodies in TBST as follows: Mouse IgG1 SHIP-1 (clone: P1C1) (Santa Cruz, Dallas, TX, USA); Rabbit Granzyme B (cell signaling, Danvers, MA, USA); and Mouse β-Actin (8H10D10) (cell signaling). Membranes were washed, then incubated with LI-COR IRDye 800CW Donkey Anti-Rabbit IgG (H + L) and LI-COR IRDye 680RD Donkey Anti-Mouse IgG (H + L) in 1% milk/TBST for 1 h at room temperature, and imaged using an Odyssey imaging system.

### Statistics

For flow cytometry and qRT-PCR data analysis, the normality of the population distribution was assessed using the D’Agostino-Pearson (*n* = ~8 mice per group) or Shapiro–Wilk (*n* = <8 mice per group) normality test by GraphPad Prism 7. Significant differences between normally distributed populations were assessed using a two-tailed, unpaired *t*-test; significant differences between non-normally distributed populations were assessed using a two-tailed Mann–Whitney exact test. The subsequent *p*-values are denoted in each figure.

## Results

### Overexpression of miR-155 Promoting Enhanced CD8^+^ T-Cell Responses

**Figure 1:**
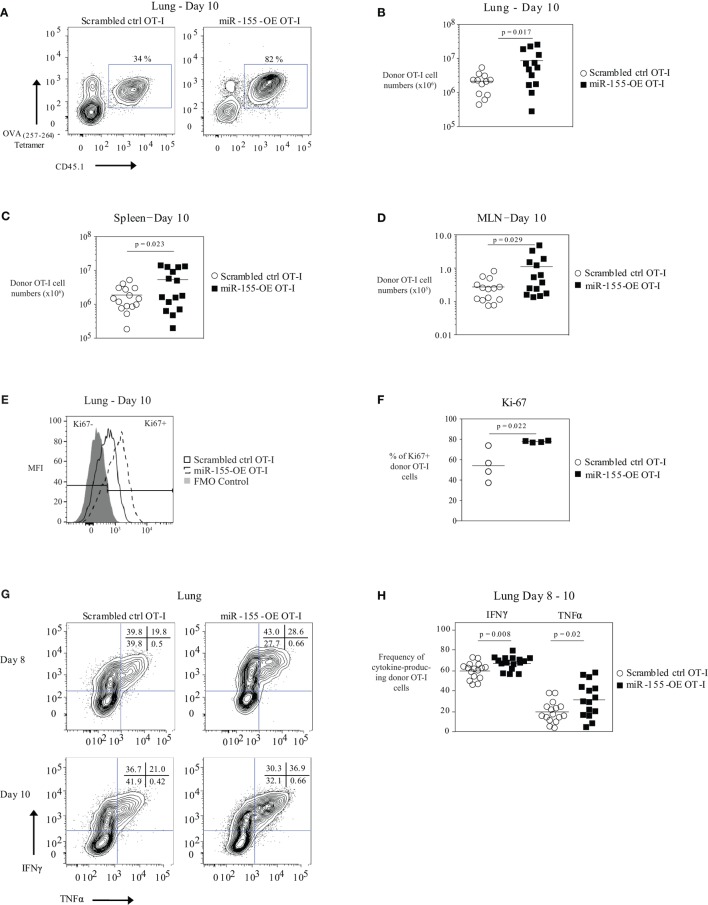
Overexpression of microRNA-155 enhancing anti-viral CD8^+^ T-cell effector expansion. **(A)** Representative flow cytometry of lungs of CD45.2^+^ wild-type mice that received CD45.1^+^ scrambled ctrl OT-I or miR-155-OE OT-I cells. Day 10 post-influenza virus infection shown. Percentages in plots indicate percent of CD45.1^+^ donor OT-I cells among lymphocytes. **(B)** Numbers of lung CD45.1^+^ donor OT-I cells for day 10 post-infection shown. Each dot represents an individual mouse, *n* = 12–13 mice per group from at least three independent experiments. Data are normally distributed (D’Agostino-Pearson) and significance was assessed using an unpaired *t*-test. Numbers of spleen **(C)** and MLN **(D)** CD45.1^+^ donor OT-I cells for day 10 post-infection shown. Each dot represents an individual mouse, *n* = 13–15 mice per group from at least three independent experiments. Data are normally distributed (D’Agostino-Pearson) and significance was assessed using an unpaired *t*-test. **(E)** Representative flow cytometry of donor OT-I cells in the lungs of influenza virus-infected animals 10 days post-infection showing gating of Ki-67^−^ and Ki-67^+^ cells. **(F)** Dot plot of the frequency of Ki-67^+^ donor OT-I cells. Each dot represents an individual mouse, *n* = 4 mice per group from one experiment. Data are normally distributed (Shapiro–Wilk) and significance was assessed using an unpaired *t*-test. **(G)** Representative IFNγ and TNFα intracellular cytokine stains of donor OT-I shown on days 8 and 10 post-influenza virus infection. Numbers in top right corner indicate percent cells in each quadrant. **(H)** Frequency of IFNγ- and TNFα-producing donor OT-I cells shown. Each dot represents an individual mouse, *n* = 15–16 mice per group from at least three independent experiments. Data are normally distributed (D’Agostino-Pearson) and significance was assessed using an unpaired *t*-test.

Previously, we demonstrated that the adoptive transfer of low numbers of miR-155-overexpressing OT-I cells resulted in increased expansion and increased viral clearance (16). To further understand the molecular basis of miR-155’s impact on CTL, we retrovirally transduced CD45.1^+^ OT-I CD8^+^ T cells to overexpress miR-155 (miR-155-OE OT-I). As a control, a retrovirus expressing a scrambled insert was used (scrambled ctrl OT-I). Transduced OT-I cells were sorted and then 10^4^ cells were adoptively transferred into CD45.2^+^ recipient mice that were then infected with WSN-OVA influenza virus. We observed a significant increase in the expansion of miR-155-OE OT-I donor cells at day 10 post-infection in the lungs of infected animals in both frequency (Figure 1A) and absolute numbers (Figure 1B), with a 2.8-fold increase in miR-155-OE OT-I donor cells by day 10 (2.1 ± 0.4 × 10^6^ versus 8.6 ± 2.3 × 10^6^, mean ± SE scrambled ctrl OT-I versus miR-155-OE OT-I, respectively, *p* = 0.017). Increased expansion of miR-155-OE OT-I CD8^+^ T cells was also observed in the spleen (1.8 ± 0.3 × 10^6^ versus 5.3 ± 1.4 × 10^6^, scrambled ctrl OT-I versus miR-155 OE OT-I, respectively, *p* = 0.023) and the mediastinal lymph nodes (MLNs) (0.2 ± 0.4 × 10^5^ versus 0.8 ± 0.3 × 10^5^, scrambled ctrl OT-I versus miR-155 OE OT-I, respectively, *p* = 0.029) 10 days post-infection (Figures 1C,D). We similarly observed a significant increase in the frequency and number of miR-155-OE donor OT-I cells in the lungs, but not in the spleen or MLN, at an earlier day 8 timepoint (Figures S1A,B in Supplementary Material). We did not observe any differences in the frequency or numbers of donor scrambled ctrl OT-I versus miR-155-OE OT-I cells in the MLN at days 5–6 post-infection indicating similar engraftment of donor cells (Figure S1C in Supplementary Material).

We did not observe any differences in the activation state (as assessed by CD25 and CD69 expression frequency and MFI) of donor OT-I when miR-155 was overexpressed (Figure S1D in Supplementary Material). Ki-67 staining of donor OT-I cells in the lungs at day 10 post-influenza virus infection showed that a greater frequency of miR-155-OE OT-I cells were Ki-67^+^ compared with scrambled ctrl OT-I cells, indicating that miR-155 overexpression enhanced the proliferation of anti-viral CD8^+^ T cells (*p* = 0.022) (Figures 1E,F). A higher frequency of miR-155-OE OT-I donor cells produced the effector cytokines IFNγ (60.3 ± 2.0% versus 67.7 ± 1.6%, mean ± SE donor scrambled ctrl OT-I versus miR-155-OE OT-I, respectively, *p* = 0.008) and TNFα (19.4 ± 2.5% versus 31.9 ± 4.4%, for donor scrambled ctrl OT-I versus miR-155-OE OT-I, respectively, *p* = 0.02) after peptide stimulation for 6 h (Figures 1G,H). No significant difference was observed in the MFI of cytokine production (data not shown). qRT-PCR analysis of the cytotoxic molecules granzyme A, granzyme B, and perforin assessed in lung donor OT-I cells 9 days post-influenza virus infection showed no difference between scrambled ctrl OT-I and miR-155-OE OT-I (Figure S1E in Supplementary Material). Furthermore, western blot analysis of granzyme B protein levels confirmed no difference (Figure S1F in Supplementary Material). These findings demonstrate that increased miR-155 expression is sufficient to promote significant expansion and increased effector cytokine production of CTL during an acute influenza virus infection.

### Overexpression of miR-155 Skewing CD8^+^ T Cells Toward an Effector Memory Phenotype

**Figure 2:**
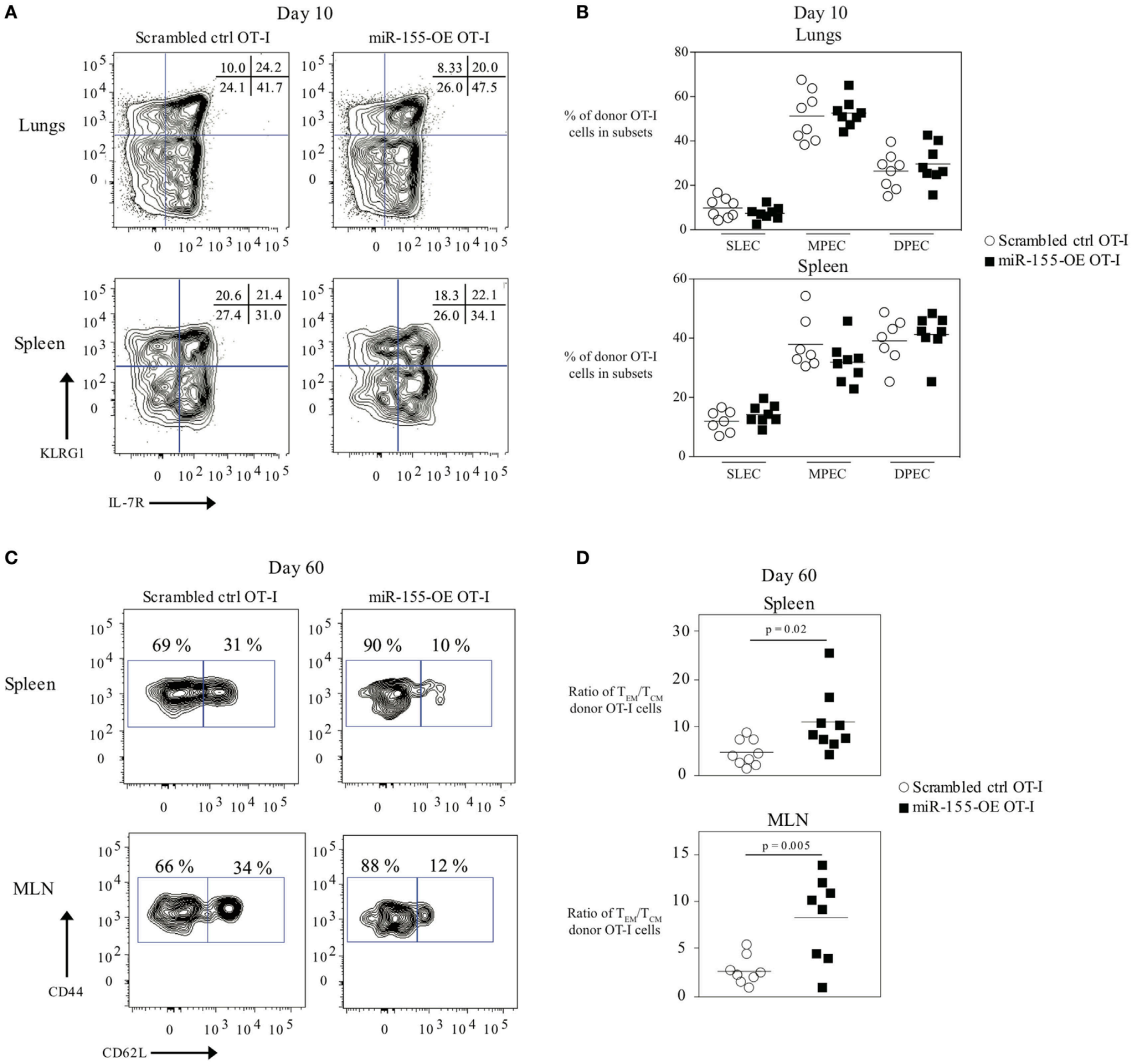
Overexpression of microRNA-155 promoting effector memory formation. **(A)** Representative flow cytometry of CD45.1^+^ donor OT-I cells from lungs or spleens of CD45.2^+^ wild-type mice that received CD45.1^+^ scrambled ctrl OT-I or miR-155-OE OT-I cells showing SLEC (KLRG1^+^IL-7R^−^) and MPEC (KLRG1^−^IL-7R^+^) populations. Numbers in plots indicate percent of CD45.1^+^ donor OT-I cells in those populations. **(B)** Frequency of SLEC, MPEC, and DPEC (double-positive effector cells) donor OT-I cells 10 days post-infection in the lungs (top) or spleen (bottom) of recipient mice. Each dot represents an individual mouse, *n* = 7–8 mice per group from two independent experiments. Data are normally distributed (D’Agostino-Pearson) and significance was assessed using an unpaired *t*-test. **(C)** Representative flow cytometry of CD45.1^+^ donor OT-I cells from spleens or MLN of CD45.2^+^ wild-type mice that received CD45.1^+^ scrambled ctrl OT-I or miR-155-OE OT-I cells day 60 after infection with influenza virus. Numbers in plots indicate percent of CD45.1^+^ donor OT-I cells in the T_EM_ (CD44^+^ CD62L^−^) and T_CM_ (CD44^+^ CD62L^+^) compartments. **(D)** Ratio of the absolute number of T_EM_/T_CM_ donor OT-I cells in the spleen (top) and MLN (bottom) shown. Each dot represents an individual mouse, *n* = 8–9 mice per group from two independent experiments. Data are normally distributed (D’Agostino-Pearson) and significance was assessed using an unpaired *t*-test.

Because miR-155 affected effector CTL responses, we also assessed its effect on memory CTL formation. We did not observe any differences in the frequencies of SLEC (short-lived effector cell) or MPEC (memory precursor effector cell) populations in donor OT-I cells in the lung or spleen 10 days post-infection (Figures 2A,B). We also did not observe differences in the frequencies or absolute numbers of donor memory OT-I CD8^+^ T cells in the lungs, spleens, or MLN (Figure S2A in Supplementary Material) when comparing miR-155-OE OT-I CD8^+^ T cells to scrambled ctrl OT-I CD8^+^ T cells. However, upon examination of the memory phenotype, there was skewing of spleen and MLN memory miR-155-OE OT-I CD8^+^ T cells toward the effector memory (T_EM_) phenotype (CD44^+^ CD62L^−^) compared with the central memory (T_CM_) phenotype (CD44^+^ CD62L^+^) (Figure 2C). The ratios of the numbers of T_EM_/T_CM_ donor OT-I CD8^+^ T cells in the spleen (*p* = 0.02) and MLN (*p* = 0.005) were skewed to the T_EM_ phenotype (Figure 2D) and a significantly lower frequency of T_CM_ donor cells in the spleen was observed (*p* = 0.004) (Figure S2B in Supplementary Material). We observed no difference in the frequency of CD44^+^ CD62L^−^CD69^+^ CD103^+^ tissue resident memory (T_RM_) OT-I populations in the lungs of recipient mice 60 days post-influenza infection (Figure S2C in Supplementary Material). Unlike at day 10, we did not observe any difference in the frequency of IFNγ- or TNFα-producing donor memory miR-155-OE OT-I CD8^+^ T cells at day 60 (Figure S2D in Supplementary Material). However, we did observe an increased expression of CD107a (LAMP-1) (Figure S2D in Supplementary Material), which indicated increased degranulation of memory miR-155-OE OT-I CD8^+^ T cells.

### T-Bet Expression in CTL Regulated by miR-155

**Figure 3:**
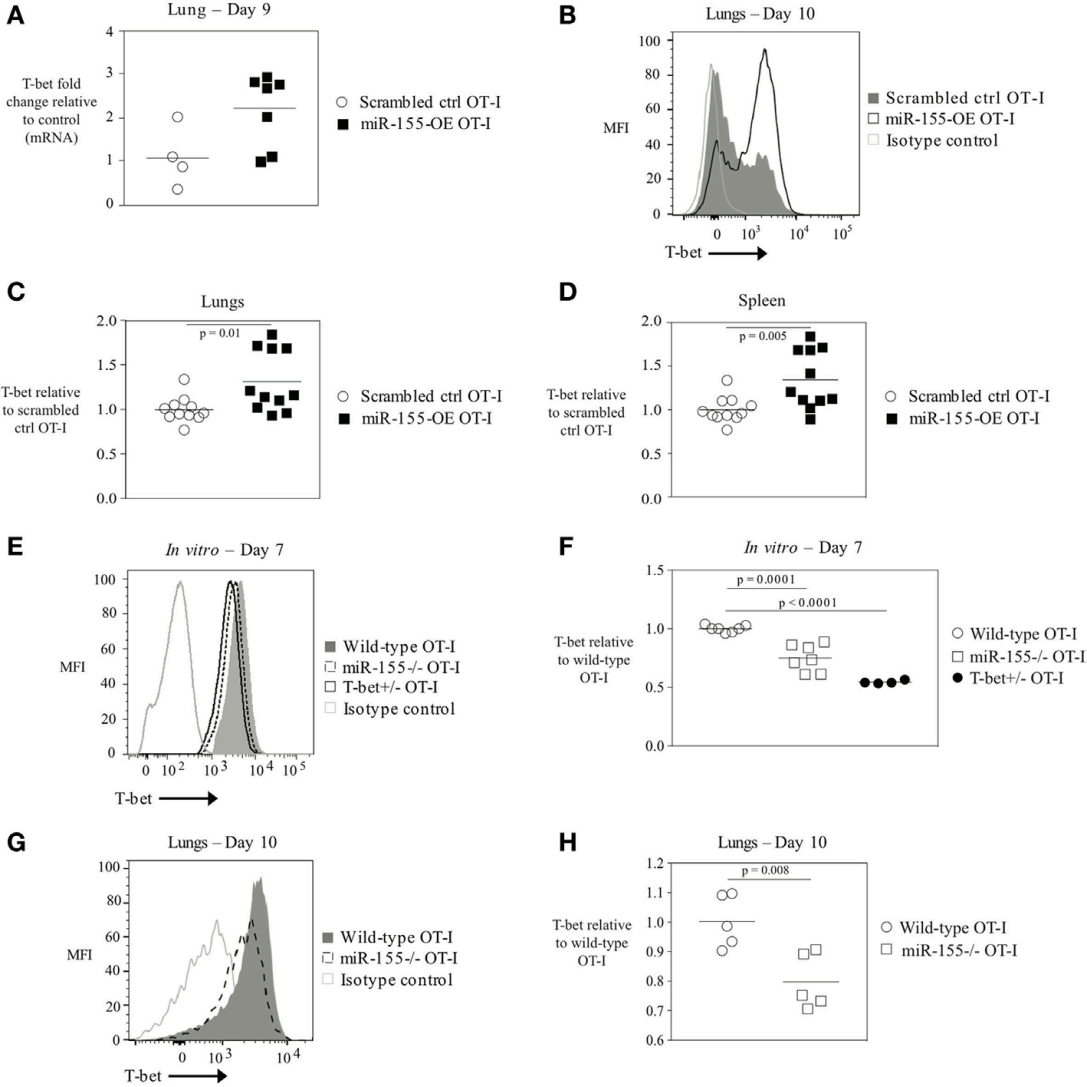
The transcription factor T-bet upregulated in miR-155-overexpressing anti-viral CD8^+^ T cells. **(A)** RT-PCR analysis of T-bet expression in donor OT-I cells sorted from lungs of WSN-OVA influenza virus-infected mice 9 days post-infection; results were normalized to GAPDH RNA expression and are presented relative to scrambled ctrl OT-I levels. Each dot represents an individual mouse, *n* = 4–7 mice per group from two independent experiments. Data are not normally distributed (Shapiro–Wilk) and significance was assessed using an unpaired Mann–Whitney exact test. **(B)** T-bet intracellular flow cytometry of donor cells from lungs of wild-type mice that received scrambled ctrl OT-I or miR-155-OE OT-I cells and assessed at day 10 after infection with influenza virus. Histogram of T-bet expression in donor OT-I cells shown is representative of at least three independent experiments. **(C)** Dot plot of T-bet expression in miR-155-OE OT-I cells relative to scrambled ctrl OT-I expression in donor cells from lungs of influenza virus-infected animals 9–10 days post-infection. Data are not normally distributed (D’Agostino-Pearson) and significance was assessed using an unpaired Mann–Whitney exact test. **(D)** Dot plot of T-bet expression in miR-155-OE OT-I cells relative to scrambled ctrl OT-I expression in donor cells in the spleen of influenza virus-infected animals 9–10 days post-infection. Data are normally distributed (D’Agostino-Pearson) and significance was assessed using an unpaired *t*-test. **(E)** Representative intracellular T-bet flow cytometry of OT-I, miR-155^−/−^ OT-I, or T-bet^+/−^ OT-I cells activated for 7 days *in vitro*. **(F)** T-bet MFI levels in *in vitro* activated OT-I, miR-155^−/−^ OT-I, or T-bet^+/−^ OT-I cells relative to OT-I control cells. Each dot represents an individual mouse, *n* = 4–7 mice per group from three independent experiments. Data are normally distributed (Shapiro–Wilk) and significance was assessed using an unpaired *t*-test. **(G)** Representative histogram and **(H)** dot plots of T-bet expression in donor OT-I and miR-155^−/−^ OT-I cells in the lungs of wild-type mice 10 days after infection with influenza virus. Data from two independent experiments, *n* = 5 animals per group. Data are normally distributed (Shapiro–Wilk) and significance was assessed using an unpaired *t*-test.

The transcription factor T-bet has previously been associated with IFNγ production, and effector and effector memory cell generation in T cells during LCMV or *L. monocytogenes* infection (6, 25). We therefore examined whether miR-155 overexpression in CD8^+^ T cells affected T-bet levels. *Tbx21* (T-bet) mRNA levels were assessed by qRT-PCR in total RNA from scrambled ctrl OT-I and miR-155-OE OT-I cells sorted from the lungs on day 9 post-infection. This demonstrated a trending increase in relative T-bet mRNA levels in miR-155-OE OT-I CD8^+^ T cells (Figure 3A). To assess T-bet expression at the protein level, T-bet expression was measured using intracellular flow cytometry that was first validated by staining T-bet^+/+^ and T-bet^+/‒^ OT-I CD8^+^ T cells, which as expected (3) demonstrated half of the amount of T-bet after both *in vitro* (Figure S3A in Supplementary Material) and *in vivo* activation (Figures S3B,C in Supplementary Material). We found that day 10 *in vivo* miR-155-OE OT-I CD8^+^ T cells express more T-bet protein compared with scrambled ctrl OT-I in the lungs (*p* = 0.01) (Figures 3B,C) and spleen (*p* = 0.005) (Figure 3D), indicating that T-bet expression in miR-155-OE OT-I CD8^+^ T cells may be regulated at the translational level as a significant difference was observed only at the protein level and not at the level of mRNA.

Since miR-155-overexpressing CTLs have increased amounts of T-bet, we reasoned that conversely miR-155-deficient CD8^+^ T cells would express decreased levels of T-bet. To assess this, splenic miR-155^−/−^ OT-I CD8^+^ T cells were isolated and activated *in vitro* with plate-bound α-CD3 and α-CD28 antibodies and cultured for 7 days. In parallel, wild-type T-bet-sufficient OT-I CD8^+^ T cells and T-bet heterozygote (T-bet^+/‒^) OT-I CD8^+^ T cells were similarly cultured. We found that T-bet^+/‒^ OT-I cells exhibited a gene-dosage effect and expressed half the amount of T-bet compared with wild-type OT-I cells (Figures 3E,F). T-bet staining of miR-155^−/−^ OT-I CD8^+^ T cells revealed a 40% (*p* = 0.0001) reduction in T-bet expression relative to wild-type OT-I CD8^+^ T cells (Figure 3F). Adoptively transferred miR-155^−/−^ OT-I cells exhibited significantly decreased T-bet levels compared with wild-type OT-I cells 10 days post-influenza virus infection (*p* = 0.008) (Figures 3E,G,H). These data provide the first evidence that miR-155 regulates T-bet expression in CTL. Furthermore, miR-155-overexpressing anti-viral CD8^+^ T cells demonstrate increased levels of T-bet, and thus enhanced T-bet expression may be responsible for the augmented effector generation and effector memory phenotype observed.

**Figure 4:**
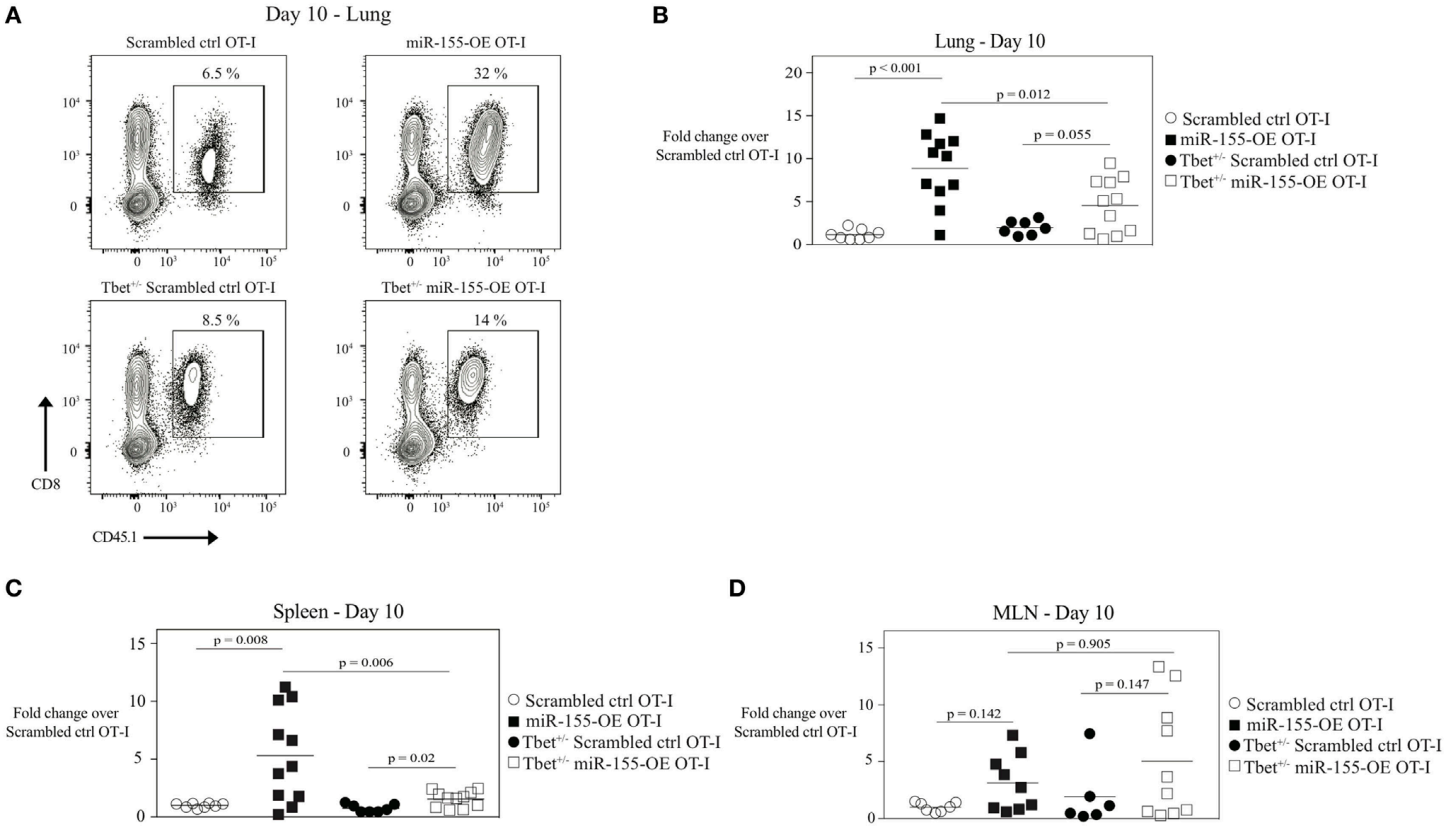
MicroRNA-155-mediated expansion of anti-viral CTL mediated by T-bet. **(A)** Representative flow cytometry of lungs of CD45.2^+^ wild-type mice that received CD45.1^+^ scrambled ctrl or miR-155-OE transduced T-bet^+/−^ or wild-type OT-I cells. Day 10 post-influenza virus infection shown. Percentages in plots indicate percent of CD45.1^+^ donor OT-I cells among lymphocytes. Dot plot showing fold-change in donor OT-I cell numbers relative to scrambled ctrl OT-I cell numbers in the lungs **(B)**, spleen **(C)**, or MLN **(D)** of recipient mice 10 days post-infection with influenza virus. Data are normally distributed (D’Agostino-Pearson) and significance was assessed using an unpaired *t*-test.

To directly test if increased T-bet in miR-155-OE OT-I cells was responsible for augmenting effector generation of anti-viral CTL, T-bet^+/‒^ OT-I or wild-type T-bet^+/+^ OT-I CD8^+^ T cells were retrovirally transduced to overexpress miR-155 or the scrambled sequence. Transduced cells were then adoptively transferred into host mice followed by infection with WSN-OVA influenza virus. miR-155-overexpressing T-bet^+/‒^ OT-I CD8^+^ T cells (T-bet^+/‒^ miR-155-OE OT-I) were incapable of expanding to the same capacity as miR-155-OE OT-I at day 10 post-infection in the lungs of influenza virus-infected animals (*p* = 0.012) (Figures 4A,B). A similar effect was observed in the spleen (*p* = 0.006), but not the MLN (Figures 4C,D). These data demonstrate that T-bet is essential to drive the increased expansion of miR-155-overexpressing CD8^+^ T cells during influenza virus infection.

### Overexpression of miR-155 Altering the Transcription Factor Profile of Memory CD8^+^ T Cells Enhancing the Expression of T-Bet and Blimp-1

**Figure 5:**
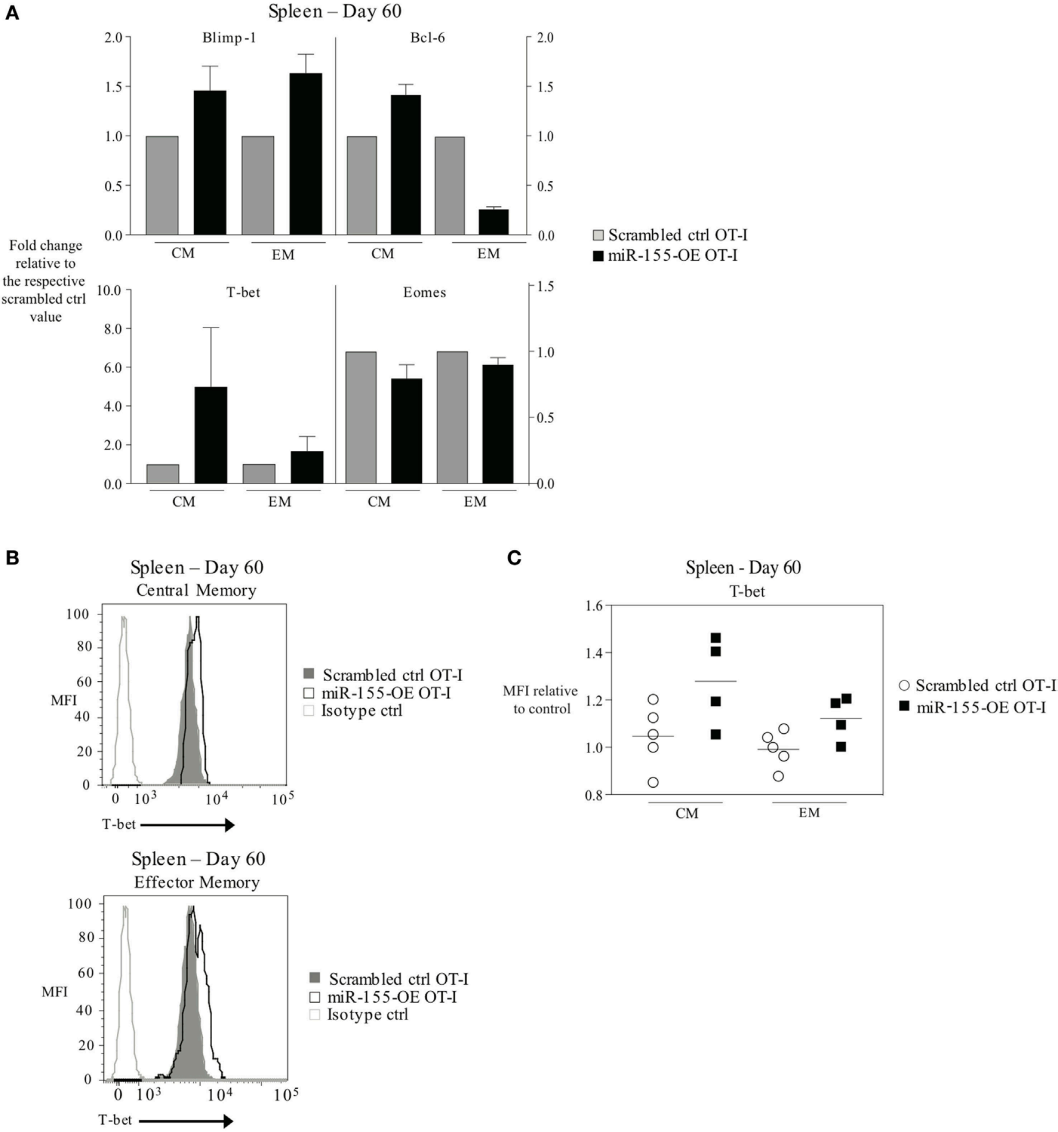
Overexpression of microRNA-155 altering the transcription factor profile of memory anti-viral CTL. **(A)** RT-PCR analysis of Blimp-1, Bcl-6, T-bet, and Eomes expression in T_CM_ (CD44^+^ CD62L^+^) or T_EM_ (CD44^+^ CD62L^−^) CD45.1^+^ CD8^+^ OT-I donor cells sorted from the spleens of CD45.2^+^ wild-type mice 60 days post-infection with WSN-OVA influenza virus. Expression levels are represented relative to the scrambled ctrl OT-I expression level within each subset. Data from two independent experiments, *n* = 2–3 mice per group. **(B)** Representative histograms and **(C)** dot plots of intracellular T-bet flow cytometry of CD44^+^ CD62L^+^ T_CM_ and CD44^+^ CD62L^−^ T_EM_ cells in spleens of wild-type mice that received scrambled ctrl OT-I or miR-155-OE OT-I cells. Day 60 after infection with influenza virus shown. Data from two independent experiments, *n* = 4–5 animals per group. T-bet expression is shown relative to scrambled ctrl OT-I.

Since transcription factors such as T-bet, Eomes, Blimp-1, and Bcl-6 are important regulators of CD8^+^ T-cell memory generation (4) and miR-155 overexpression promoted effector memory CTL formation, we examined the transcription factor profile of miR-155-overexpressing memory CTL. To assess this, T_CM_ and T_EM_ donor cell compartments were sorted 60 days post-influenza virus infection and mRNA expression levels of *Tbx21* (encoding T-bet), *Eomes, Prdm1* (encoding Blimp-1), and *Bcl6* were measured. We observed that Blimp-1 mRNA expression was modestly increased in miR-155-OE T_EM_ OT-I cells, while Bcl-6 mRNA expression was decreased in miR-155-OE T_EM_ OT-I cells compared with scrambled ctrl OT-I CD8^+^ T cells (Figure 5A, top). Interestingly, we observed in both T_EM_ and T_CM_ compartments an increase in T-bet mRNA expression when miR-155 was overexpressed, with the greatest increase observed in the T_CM_ compartment (Figure 5A, bottom). We did not observe any differences in Eomes mRNA expression. Intracellular staining and flow cytometry confirmed increased T-bet expression when miR-155 was overexpressed in both T_EM_ and T_CM_ compartments in the spleen (Figures 5B,C).

### miR-155 Regulating SHIP-1 Expression in Anti-Viral CD8^+^ T Cells Modulating T-Bet Levels

**Figure 6:**
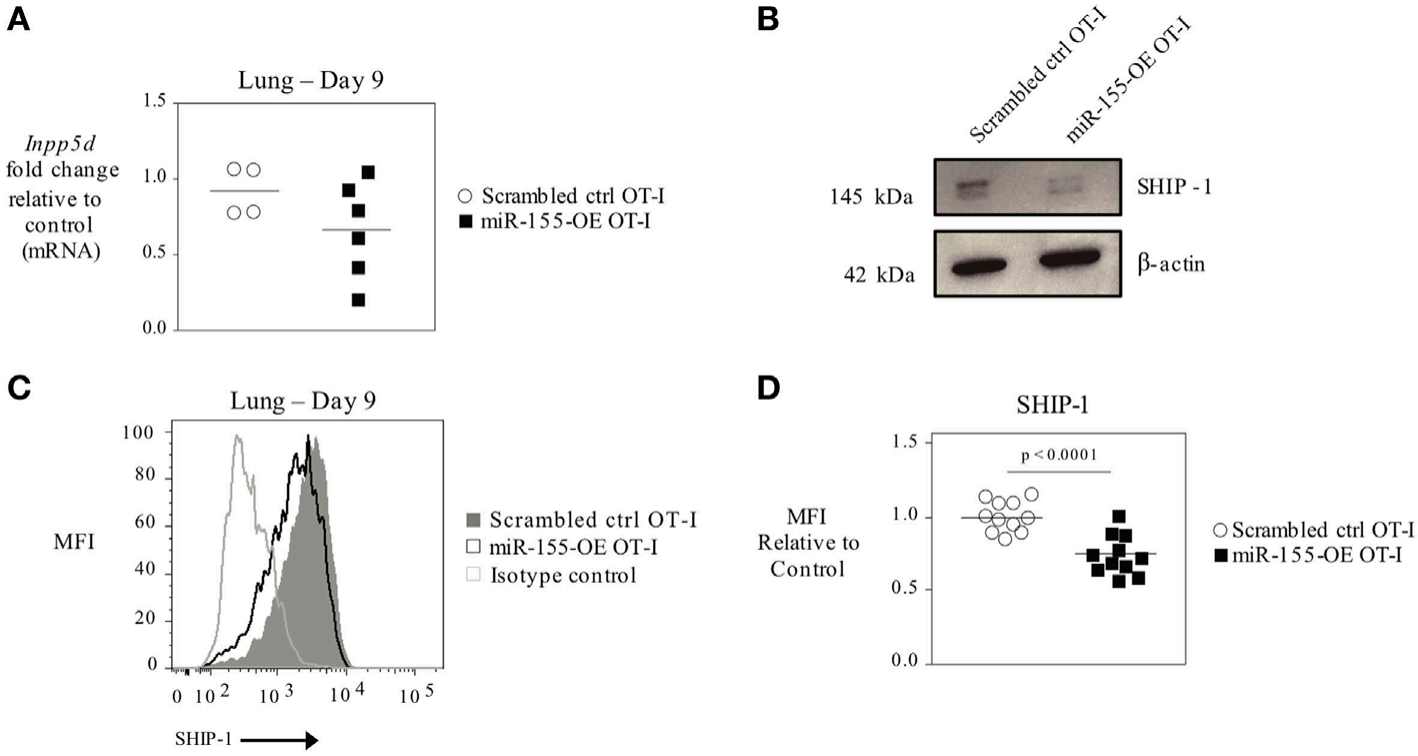
SHIP-1 downregulated by miR-155 in anti-viral CTL. **(A)** RT-PCR analysis of SHIP-1 levels in donor OT-I cells sorted from lungs 9 days post-infection with WSN-OVA influenza virus. Results were normalized to GAPDH RNA expression and are presented relative to scrambled ctrl OT-I expression levels. Each dot represents an individual mouse, *n* = 4–6 mice per group from two independent experiments. Data are normally distributed (Shapiro–Wilk) and significance was assessed using an unpaired *t*-test. **(B)** Western blot analysis of SHIP-1 expression in sorted donor scrambled ctrl or miR-155-OE OT-I cells. Representative of three mice per group. **(C)** Representative histogram of intracellular SHIP-1 flow cytometry of donor OT-I cells from lungs 9–10 days post-infection with influenza virus. Histograms gated on CD45.1^+^ donor cells. **(D)** SHIP-1 MFI expression levels relative to scrambled ctrl OT-I expression levels. Each dot represents an individual mouse, *n* = 11 mice per group from three independent experiments. Data are normally distributed (D’Agostino-Pearson) and significance was assessed using an unpaired *t*-test.

**Figure 7:**
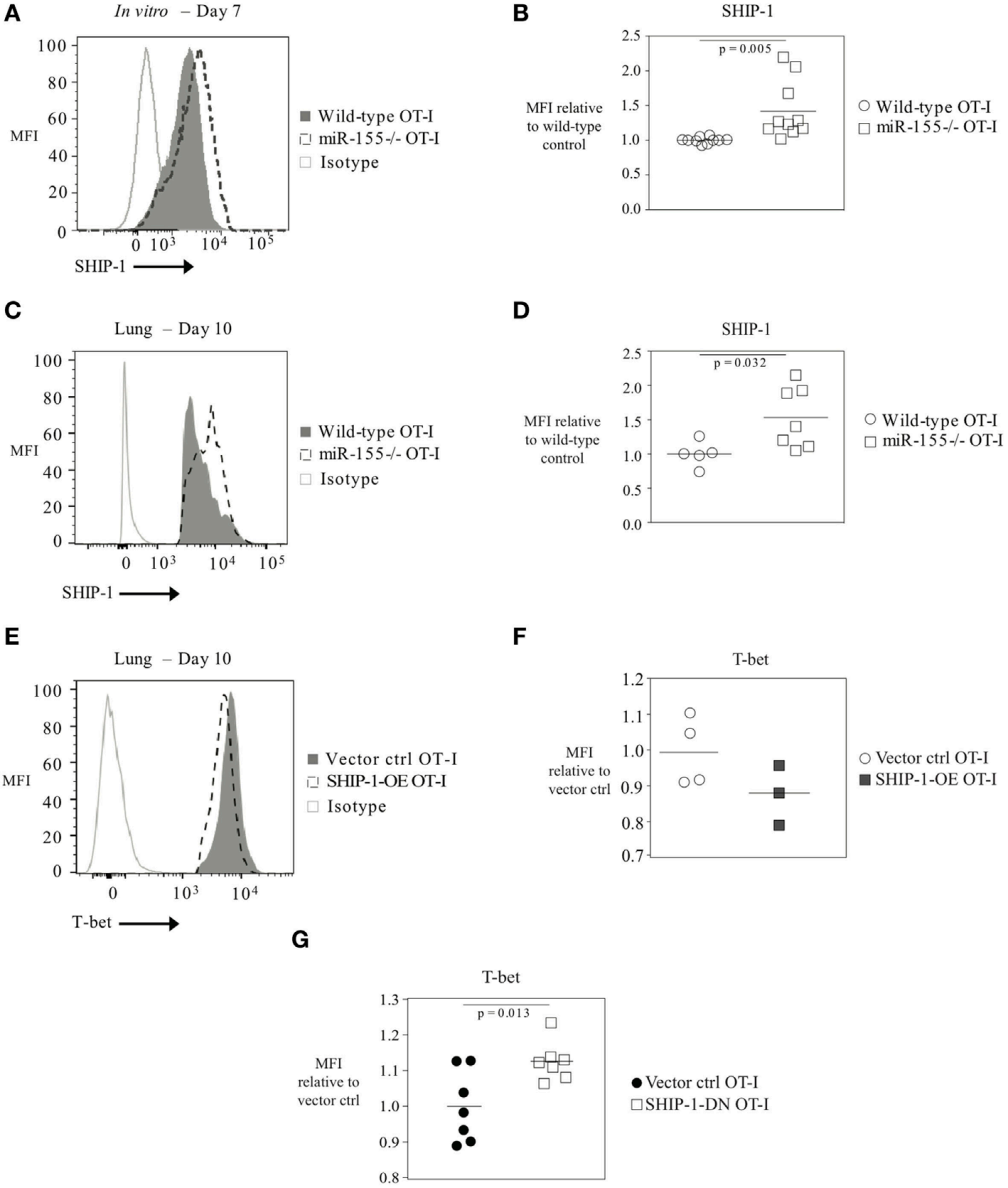
T-bet expression regulated by SHIP-1 in anti-viral CTL. **(A)** Representative histogram of intracellular SHIP-1 flow cytometry of OT-I or miR-155^−/−^ OT-I cells activated for 7 days *in vitro* with solid plate-bound α-CD3/α-CD28 antibodies. **(B)** SHIP-1 MFI levels in *in vitro* activated OT-I or miR-155^−/−^ OT-I cells relative to OT-I control cells. Each dot represents an individual mouse, *n* = 10 mice per group from four independent experiments. Data are normally distributed (D’Agostino-Pearson) and significance was assessed using an unpaired *t*-test. **(C)** Representative histogram and **(D)** dot plots of intracellular SHIP-1 flow cytometry of lung donor OT-I or miR-155^−/−^ OT-I cells on day 10 post-infection with influenza virus. Data from *n* = 5–7 mice per group and two independent experiments shown. Data are normally distributed (Shapiro–Wilk) and significance was assessed using an unpaired *t*-test. **(E)** Representative histograms and **(F)** dot plots of intracellular T-bet flow cytometry of lung vector ctrl OT-I or SHIP-1-OE OT-I cells on day 10 post-influenza virus infection. T-bet MFI expression levels relative to vector ctrl OT-I expression levels. Each dot represents an individual mouse, *n* = 3–4 mice per group from two independent experiments. Data are normally distributed (Shapiro–Wilk) and significance was assessed using an unpaired *t*-test. **(G)** T-bet MFI expression levels in SHIP-1DN or vector-transduced donor OT-I CD8^+^ T cells 10 days post-infection with WSN-OVA influenza virus in the spleen. Expression is shown relative to vector ctrl OT-I expression levels. Each dot represents an individual mouse, *n* = 7 mice per group from two independent experiments. Data are normally distributed (Shapiro–Wilk) and significance was assessed using an unpaired *t*-test.

As T-bet is not predicted to be a direct target of miR-155, we reasoned that miR-155 was regulating T-bet expression indirectly. SHIP-1 has previously been identified as a direct target of miR-155 and has been reported to be affected by miR-155 in CTL (16, 20, 26). We assessed SHIP-1 expression at both the mRNA and protein levels when miR-155 is overexpressed in CTL. qRT-PCR of sorted day 9 post-influenza virus infection miR-155-OE OT-I cells showed a 25% decrease in SHIP-1 (*Inpp5d)* mRNA expression levels (Figure 6A) compared with controls. Western blot analysis (Figure 6B) and intracellular flow cytometry staining (Figures 6C,D) of miR-155-OE OT-I CD8^+^ T cells revealed that miR-155 overexpression resulted in significantly decreased (*p* < 0.0001) SHIP-1 protein expression in anti-viral CTL on days 9–10 post-influenza virus infection, demonstrating that like T-bet, SHIP-1 expression is modulated at the translational level in miR-155-OE CD8^+^ T cells. As miR-155 overexpression led to decreased SHIP-1 protein expression and microarray analysis had previously identified a modest (1.20-fold, *p* = 0.01) upregulation of SHIP-1 mRNA in miR-155-deficient CTL after 3 days of *in vitro* activation (16), we sought to assess SHIP-1 protein expression in miR-155-deficient CTL. Intracellular flow cytometry confirmed that miR-155^−/−^ OT-I cells cultured *in vitro* for 7 days expressed significantly more SHIP-1 compared with wild-type control cells (1.45-fold increase of MFI, *p* = 0.005) (Figures 7A,B). Adoptively transferred miR-155^−/−^ OT-I cells also demonstrated significantly increased SHIP-1 levels compared with wild-type OT-I cells 10 days post-infection with influenza virus (*p* = 0.032) (Figures 7C,D). Finally, we tested if SHIP-1 directly mediates the effect of miR-155 on T-bet expression. OT-I cells were transduced with either an SHIP-1-overexpressing retrovirus (SHIP-1-OE OT-I) or a dominant-negative SHIP-1 resulting in the inhibition of SHIP-1 signaling (23) (SHIP-1DN OT-I) and adoptive transfers were performed. We show that SHIP-1 overexpression alone is sufficient to decrease T-bet relative to control levels (Figures 7E,F), while SHIP-1-DN results in significantly increased T-bet expression levels in donor OT-I cells (*p* = 0.013) (Figure 7G). Taken together, these data provide further support that miR-155 modulates T-bet expression *via* SHIP-1.

## Discussion

The importance of miRNA in T cells, particularly CD8^+^ T cells, has been demonstrated with Dicer-deficient animals (12, 13, 27), where the lack of mature miRNA has profound effects on the differentiation and function of T cells. Since miR-155 is upregulated in effector CTL and we have previously shown that the absence of miR-155 significantly reduces effective CTL responses to infection (16), we argued that its forced overexpression would enhance the CTL response. Indeed, overexpression of miR-155 in CTL augmented the response, in agreement with previous reports from us and others (16, 17, 20). In exploring the mechanistic underpinnings of the effect of miR-155 overexpression, we have revealed an unexpected connection between miR-155 and T-bet expression in virus-specific CD8^+^ T cells, mediated through the SHIP-1-signaling pathway. miR-155 has been shown, by us and others, to control the CTL immune response (14, 16–20), while the direct targets by which miR-155 affects CTL responses have been suggested to be context dependent (14). Previous studies have indicated that miR-155 in CD8^+^ T cells may regulate SOCS-1 and STAT5 in acute and chronic LCMV infection (17) and tumors (17, 20), STAT1/STAT2 and type I IFN signaling in influenza virus infection (16), and c-maf in CD4^+^ T cells (28) depending on the cell type and the environment in which the response is taking place. To the best of our knowledge, this is the first report that miR-155 can regulate T-bet expression in CTL and miR-155 overexpression leads to increased numbers of effector CTL and a skewing of CTL memory to an effector memory phenotype. This is consistent with T-bet’s well-known capacity to promote effector CTL expansion and effector memory CD8^+^ T-cell generation. Indeed, we find that the increased effector CTL expansion is induced by miR-155 overexpression and is mediated by T-bet as miR-155-overexpressing T-bet^+/‒^ CTL fail to expand.

In CD8^+^ T cells, T-bet promotes the differentiation of effector CTL and is associated with T_EM_ CD8^+^ T cells (3). In the absence of T-bet during a viral infection such as LCMV, CTL differentiation was skewed toward a KLRG1^lo^CD127^hi^ memory precursor phenotype with less formation of terminally differentiated effector cells (3), and memory CD8^+^ T cells are maintained (29). Overexpression of T-bet sustains effector CTL during chronic LCMV infection (30). Because T-bet mRNA 3′UTR lacks a miR-155 seeding sequence and is not a known or predicted target of miR-155, we postulated that this is an indirect effect. Indeed, we report that the modulation of T-bet levels by miR-155 is mediated by miR-155’s direct target SHIP-1. The relationship between SHIP-1 and T-bet has been previously demonstrated by Tarasenko et al. who observed that SHIP-1-deficient CD8^+^ T cells expressed 60% more T-bet than control CD8^+^ T cells (31). This raises the question of how SHIP-1 signaling negatively affects T-bet levels. One pathway that is well established to control T-bet expression is mTOR signaling (8), which is downstream of phosphatidylinositol-3-kinase (PI3K) signaling (32). SHIP-1 is known to inhibit PI3K signaling (33), which would result in reduced mTOR activity and decreased T-bet. miR-155, by suppressing SHIP-1 levels, may amplify PI3K and mTOR signaling, thus resulting in more T-bet. Future studies examining the PI3K pathway when miR-155 is modulated could address this question.

Here, we report that SHIP-1 can be downregulated in CTL as a result of miR-155 overexpression, while miR-155 deficiency leads to increased SHIP-1 levels. SHIP-1 is a bona fide direct target of miR-155 and this downregulation has been shown to play an important functional role in many immune cell subsets including macrophages (26), NK cells (34), and dendritic cells (35) and SHIP-1 levels have been reported to be modulated in CTL by miR-155 overexpression (20). In our current study, we demonstrate that this intrinsic SHIP-1 modulation impacts CTL responses. Although the role of SHIP-1 in immune cells has been extensively studied, the role of SHIP-1 in T cells is less clear. There is conflicting data regarding how SHIP-1 expression impacts the ratio of peripheral CD4^+^ and CD8^+^ T-lymphocyte populations (31, 36), and the role of SHIP-1 in *in vivo* CTL responses is questionable. In a previous study by Tarasenko et al., the role of SHIP-1 in T cells was explored using a CD4^cre^ SHIP^fl/fl^ mice in which all T cells lacked SHIP-1 expression. Although a Th1 skewing of CD4^+^ T cells was observed, no differences were found in the *in vivo* expansion of SHIP-deficient CD4^+^ or CD8^+^ T cells in mice immunized with ovalbumin (31). However, SHIP-1 may play an important role in CTL in the inflammatory milieu of a viral infection, where dampening inflammatory signaling pathways may be critical for the CTL responses. Alternatively, the effect of SHIP-1 on CTL that overexpress miR-155 may be due to additional miR-155-mediated perturbations in other signaling pathways that change the balance between signaling pathways and raise SHIP-1 to a more prominent functional role.

We find that miR-155 overexpression also affects memory CTL responses. We found that T_EM_ CTL formation was favored by miR-155 overexpression. Indeed, we previously observed that miR-155 was more highly expressed in anti-viral T_EM_ versus T_CM_ (16). This most likely is due to miR-155 overexpression increasing T-bet expression as T-bet is known to promote T_EM_ CTL formation (8, 37). However, miR-155 overexpression also increased Blimp-1 and decreased Bcl-6 in memory cells. Thus, the promotion of T_EM_ CTL formation could be due to changes in several transcriptional factors that affect T_EM_ CTL. Interestingly, we found that miR-155-overexpressing T_CM_ CTL also increased T-bet expression in the spleen. Since the numbers of T_EM_ and T_CM_ CTL were not increased in spleens, it is possible that increased T-bet levels in T_CM_ promotes their conversion to T_EM_, a dynamic process previously observed in vesicular stomatitis virus (VSV)-specific and *L. monocytogenes*-specific memory CTL (38, 39), and leads to their preferential migration to sites of inflammation such as the lung or MLN. This is consistent with T-bet’s ability to drive T_EM_ formation but also suggests that miR-155 overexpression could regulate plasticity of CTL memory phenotypes.

We report here that miR-155 overexpression leads to increased effector CTL responses and a skewing of memory CTL toward an effector memory phenotype. To the best of our knowledge, our studies conclusively show for the first time a novel connection between miR-155 and T-bet in CD8^+^ T cells. The link between miR-155 overexpression and T-bet upregulation is essential for the expansion of effector CTL. We show that T-bet is indirectly targeted by miR-155 *via* miR-155’s repression of its direct target SHIP-1. Our studies reveal an unexpected miR-155/SHIP-1/T-bet axis in CTL immunity to viral infection that may play a pivotal role in CTL immunity in the context of infection and cancer.

## Ethics Statement

This study was carried out in accordance with the recommendations of the Institutional Animal Care and Use Committee (IACUC), Drexel University College of Medicine or the Instantie voor Dierenwelzijn (IvD), Erasmus University Medical Center. The protocols were approved by IACUC or IvD.

## Author Contributions

JH performed infections, adoptive transfers, retroviral transduction, and flow cytometry. CS, PS, AC, DG, AF, MM, IB-H, LR, and JF performed infections, flow cytometry, RT-PCR, *in vitro* assays, retroviral production, and mouse breeding. RK and SE cloned and designed vectors. YM performed adoptive transfers and cell sorting. ES, EW, JH, and PK designed and performed T-bet experiments. JH and PK were responsible for study design, data analysis, and manuscript authorship. All authors discussed the results and commented on manuscript.

## Conflict of Interest Statement

The authors declare that the research was conducted in the absence of any commercial or financial relationships that could be construed as a potential conflict of interest.
