# Supplementary material for: The Transcription Factor T-Bet Is Regulated by MicroRNA-155 in Murine Anti-Viral CD8+ T Cells via SHIP-1
Source: Front Immunol. 2017 Dec 6;8:1696. doi: 10.3389/fimmu.2017.01696 (PMC5765282; doi:10.3389/fimmu.2017.01696)
Supplement: Supplementary file 1 [file presentation_1.pdf]

S 1

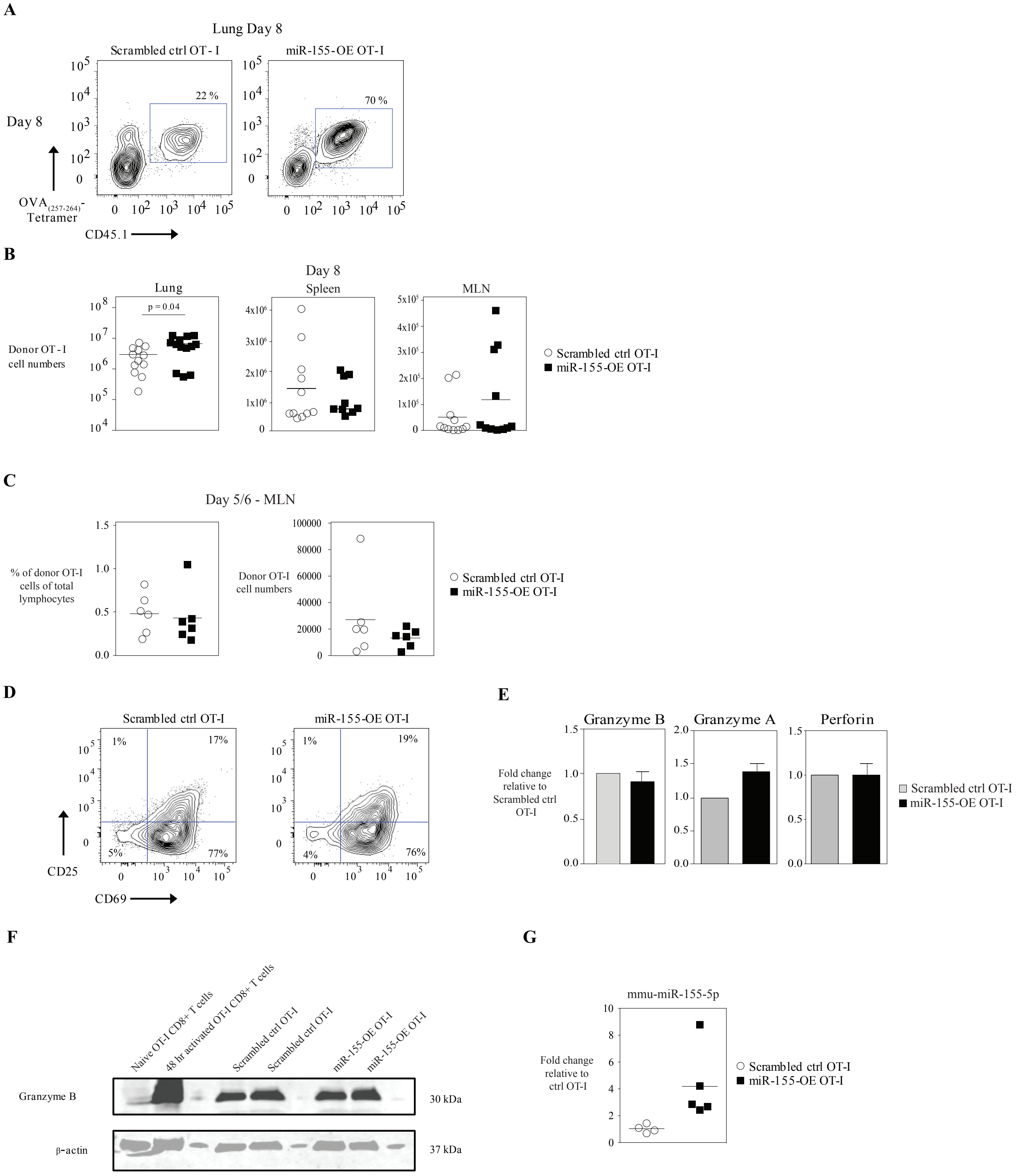
A
Lung Day 8
Scrambled ctrl OT - I
miR-155-OE OT-I
22 %
70 %
Day 8
OVA(257-264)-Tetramer
CD45.1
B
Day 8
Lung
Spleen
MLN
p = 0.04
Donor OT - I cell numbers
Scrambled ctrl OT-I
miR-155-OE OT-I
C
Day 5/6 - MLN
% of donor OT-I cells of total lymphocytes
Donor OT-I cell numbers
Scrambled ctrl OT-I
miR-155-OE OT-I
D
Scrambled ctrl OT-I
miR-155-OE OT-I
1%
17%
5%
77%
1%
19%
4%
76%
CD25
CD69
E
Granzyme B
Granzyme A
Perforin
Fold change relative to Scrambled ctrl OT-I
Scrambled ctrl OT-I
miR-155-OE OT-I
F
Naive OT-I CD8+ T cells
48 hr activated OT-I CD8+ T cells
Scrambled ctrl OT-I
Scrambled ctrl OT-I
miR-155-OE OT-I
miR-155-OE OT-I
Granzyme B
30 kDa
β-actin
37 kDa
G
mmu-miR-155-5p
Fold change relative to ctrl OT-I
Scrambled ctrl OT-I
miR-155-OE OT-I

S1. MiR-155 overexpression does not alter the activation state or cytotoxic molecules of CTL. (A) Representative flow cytometry of lungs of CD45.2+ wild-type mice that received CD45.1+ scrambled ctrl OT-I or miR-155-OE OT-I cells. Day 8 post-influenza virus infection shown. Percentages in plots indicate the frequency of CD45.1+ and $OVA_{(257\text{-}264)}$-Tetramer-specific CD8+ T cells within lymphocytes. (B) Numbers of CD45.1+ donor OT-I cells in lungs (left), spleen (center), and MLN (right) in mice 8 days post-infection. Each dot represents an individual mouse. Data is normally distributed (D'Agostino-Perason) and significance was assessed using an unpaired *t*-test. At least 9 animals per group, from at least three independent experiments. (C) Frequency (left) and number (right) of CD45.1+ donor OT-I cells in the MLN of CD45.2+ wild-type mice days 5-6 post infection with WSN-OVA influenza virus. Data from two experiments. (D) Representative flow cytometry of CD25 and CD69 expression on donor OT-I cells at 10 days post-infection with influenza virus. Representative of at least 9 animals per group, at least three independent experiments. (E) RT-PCR analysis of granzyme B, granzyme A, and perforin mRNA expression in donor OT-I cells sorted from the lungs of recipient mice 9 days post-infection with influenza virus. Scrambled ctrl OT-I values represent a pooling of 3 mice. miR-155-OE OT-I values representative of 5 mice. (F) Western blot analysis of granzyme B in sorted donor OT-I cells as in (E). Representative of two independent experiments with four mice per group. (G) RT-PCR analysis of mature mmu-miR-155-5p RNA expression in donor OT-I cells sorted from the lungs of recipient mice 9 days post-infection with influenza virus. Each dot represents an individual mouse. N =4 animals per group, two independent experiments.

S2

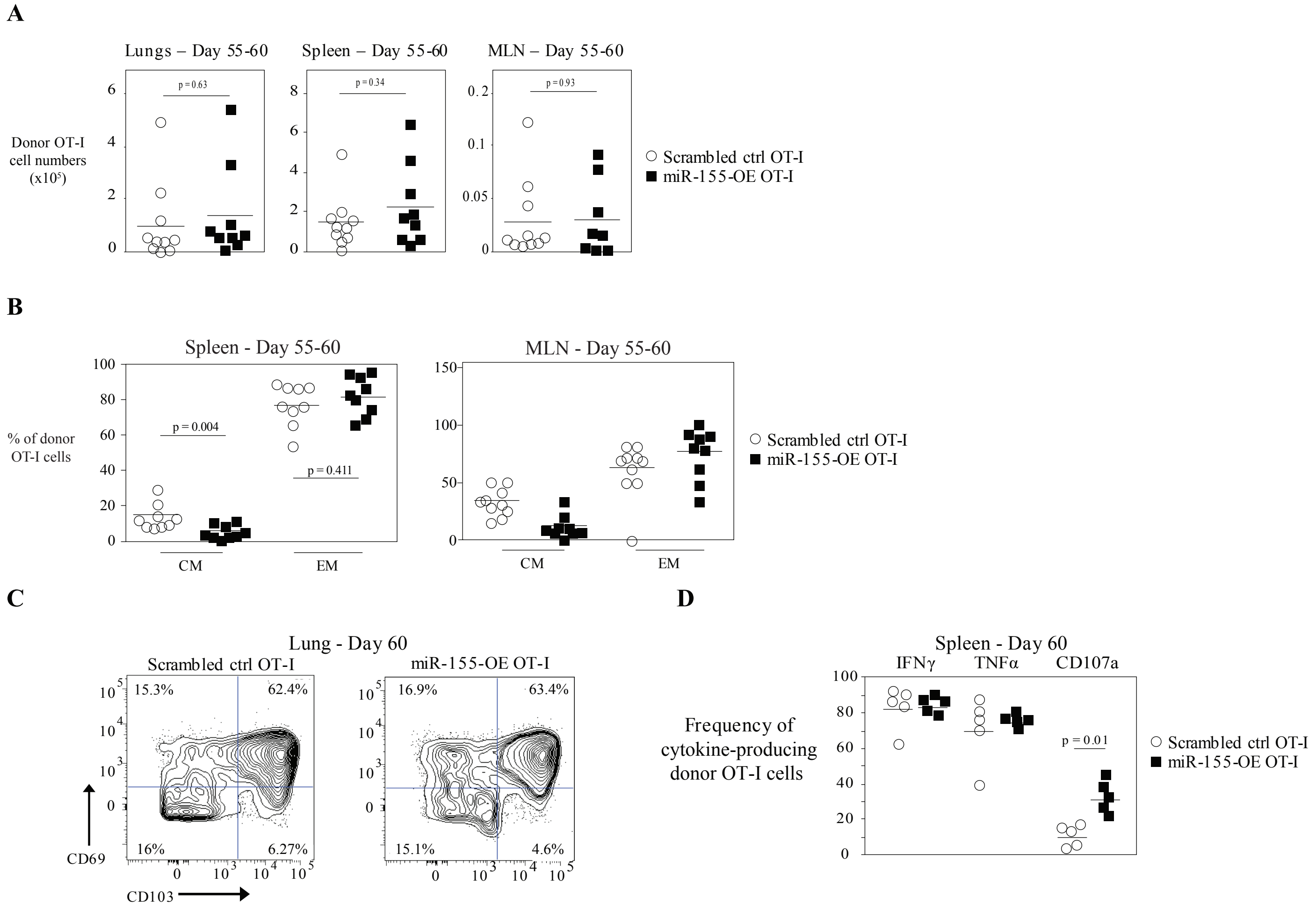
A
Lungs – Day 55-60
Spleen – Day 55-60
MLN – Day 55-60
p = 0.63
p = 0.34
p = 0.93
Donor OT-I cell numbers (x10⁵)
Scrambled ctrl OT-I
miR-155-OE OT-I
B
Spleen - Day 55-60
MLN - Day 55-60
% of donor OT-I cells
p = 0.004
p = 0.411
CM
EM
Scrambled ctrl OT-I
miR-155-OE OT-I
C
Lung - Day 60
Scrambled ctrl OT-I
miR-155-OE OT-I
15.3%
62.4%
16%
6.27%
16.9%
63.4%
15.1%
4.6%
CD69
CD103
D
Spleen - Day 60
IFNγ
TNFα
CD107a
Frequency of cytokine-producing donor OT-I cells
p = 0.01
Scrambled ctrl OT-I
miR-155-OE OT-I

S2. MiR-155 overexpression does not increase memory CTL numbers. (A) Absolute number of donor OT-I cells in the lungs (left), spleens (center), or MLN (right) of recipient mice 55-60 days post-infection with WSN-OVA. Each dot represents an individual mouse, at least 8 mice per group, data from at least 3 independent experiments. Data is normally distributed (D'Agostino-Pearson) and signficance was assessed using an unpaired *t*-test. (B) Dot plot indiciating the frequency of CM (CD44+CD62L+) and EM (CD44+CD62L-) donor OT-I cells in the spleens (left) and MLN (right) of recipient mice 55-60 days post-infection with WSN-OVA influenza virus. Each dot represents an individual mouse, 9 animals per group from three independent experiments. EM populations are normally distributed; CM populations are not. Significance was assessed using either an upaired *t*-test (EM) or Mann-Whitney exact test (CM). (C) Flow cytometry showing the frequency of T_{RM} populations in the lungs of recipient mice 60 days post-influenza infection. Pre-gated on CD8+CD45.1+ lymphocytes. Representative of two independent experiments. (D) Dot plot of the frequency of IFNγ-producing, TNFα-producing, or CD107a-expressing donor OT-I cells in the spleens of recipient mice 60 days post-infection with WSN-OVA. 5 mice per group from two independent experiments. Data is normally distributed (Shapiro-Wilk) and significance was assessed using an unpaired *t*-test.

S3

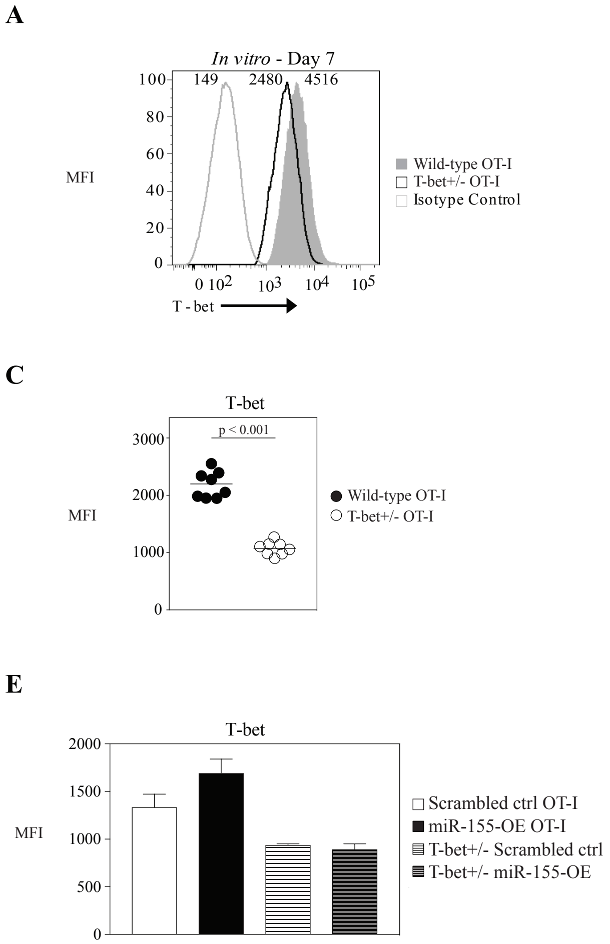
A
In vitro - Day 7
149
2480
4516
MFI
T - bet
Wild-type OT-I
T-bet+/- OT-I
Isotype Control
B
Lungs - Day 10
MFI
Wild-type OT-I
T-bet+/- OT-I
Isotype Control
C
T-bet
p < 0.001
MFI
Wild-type OT-I
T-bet+/- OT-I
D
Lungs - Day 10
MFI
Scrambled ctrl OT-I
miR-155-OE OT-I
T-bet+/- Scrambled ctrl
T-bet+/- miR-155-OE
Isotype Control
E
T-bet
MFI
Scrambled ctrl OT-I
miR-155-OE OT-I
T-bet+/- Scrambled ctrl
T-bet+/- miR-155-OE

S3. Validation of T-bet intracellular flow cyometry staining. (A) Representative histogram of T-bet expression levels in wild-type OT-I and T-bet+/- OT-I CD8+ T cells activated *in vitro* for 7 days. Numbers reflect T-bet MFI. (B) Representative histogram of T-bet expression in donor wild-type OT-I and T-bet+/- OT-I CD8+ T cells from the lungs of WSN-OVA influenza virus infected animals 10 days post-infection. (C) Dot plot of T-bet expression (MFI) of donor wild-type OT-I and T-bet+/- OT-I cells in lungs 10 days post-infection. 8 animals per group from three independent experiments. Data is normally distributed (D'Agostino-Pearson) and significance was assessed using an unpaired *t*-test. (D) Representative histogram of T-bet expression in donor scrambled ctrl OT-I, miR-155-OE OT-I, T-bet+/- scrambled ctrl OT-I, and T-bet+/- miR-155-OE OT-I compared to isotype control from the lungs of WSN-OVA influenza virus infected animals 10 days post-infection. (E) Representative bar graph of T-bet expression (MFI) of donor OT-I cells in lungs 10 days post-infection.
